# Strictures on "Official Papers Relating to Operations Performed by Mr. Adams for the Cure of Cataract and the Egyptian Ophthalmia," with Remarks

**Published:** 1814-12

**Authors:** C. W. Smerdon

**Affiliations:** Member of the College of Surgeons. Clifton, Bristol


### *For the Medical and Physical Journal.*

*Strictures on "Official Papers relating to Operations performed by Mr. Adams for the Cure of Cataract and the Egyptian Ophthalmia," with Remarks;* by Mr. C. W. SMERDON, of Clifton, Bristol.

NEARLY ten months have elapsed since the directors of Greenwich Hospital presented to the public the result of Sir W. Adams's successful operations on the eyes of the blind pensioners, as drawn up by himself, and the medical officers of that great national institution. Every encouragement ought certainly to be given to those who increase the comforts of the veteran defenders of our country, and surely it is no small consideration to restore to thirty of these men the blessing of sight. Had he solely grounded his claim to fame and honour on this simple fact, of which the directors were fully competent to judge, they could have had no cause to complain of his subsequent conduct; but he accompanied the exhibition of his patients with a letter, wherein he has made use of assertions as to his mode of cure, which, for the honour of his profession, and to avoid the stigma of empiricism, he was bound to explain his practice, not indeed to the directors of Greenwich Hospital, but to the medical public, who alone can be competent judges of their merits or defects.

This letter, together with its satellite, published as they were by such high authority, have been widely disseminated among the public at large; and, as the knight has therein asserted, that his mode of operating totally differs from that practised by the most eminent oculists in London, which might be construed into an assumption of superiority, so he has contrived to throw into his scale a considerable share of popularity, to which indeed he would be entitled, were his modes of operating found by his competitors to be what he wishes them to be considered.

It is, I think, quite impossible for a medical person to read those Official Papers, as they are called, without generating ideas not quite so favourable of their authors as they in reality may deserve. The different operations on the eye are dexterously glanced over in such a manner as to throw on them an obscure light, just sufficient to render darkness visible, and to make obscurity still more obscure. In the title-page the cure of the Egyptian ophthalmia is coupled with that of the cataract, which, singular to say, (as I shall presently endeavour to prove) is founded in error: this, however, does not appear to have originated with the knight, but but from the following paragraph, which I have copied from the letter of the medical officers of Greenwich Hospital.

"In addition to the gratifying contents of the second report, we think it our duty to state, for the information of the board, that Mr. Adams has discovered a mode of curing the Egyptian ophthalmia, which has been successfully practised upon several of the pensioners, some of whom had been blind for three or four years, and given up as incurable by the most eminent oculists then in London. The communication that this destructive and hitherto intractable disease admits of cure, we conceive will be gladly received by the board, and the promulgation by Mr. Adams of this important discovery be considered as a great national desideratum."

It is only necessary to advert to the nature of the disease in question to show, that the fact must be otherwise than what is stated in this quotation: it is preposterous to suppose that the Egyptian ophthalmia could retain its original character for the space of three years (or even three days) without wearing out the vitality of the organ which it attacks, and then we should have no longer the Egyptian ophthalmia to contend with, but its destructive effects, and no doubt it is these which are alluded to in the above paragraph.

Independently of any peculiarity which may or may not exist in the soil or climate of Egypt, the Egyptian ophthalmia is clearly of the inflammatory kind, differing only in degree from the common acute ophthalmia of this country, and in the sudden violence of its attack; and hence it is that so many of our soldiers irrecoverably lost their sight, almost as soon as cognizance was taken of the disease.

The anatomical structure of the eye readily shows why this dreadful malady, when not decidedly mitigated in a very short time, should make such extensive ravages: the vessels of that part of the conjunctiva which lines the opake cornea are imbedded in a loose reticular membrane, which allows of their dilatation, as well as accommodating themselves to sudden distension when blood is driven into the eye with impetuosity, by the action of inflammation; the transparent cornea, on the contrary, has not such a structure, for it is evident that a loose cellular membrane interposed between the laminæ of this tunic, would prevent the passage of the rays of light through it; here then the conjunctiva, together with its vessels, (which are continuous with those just mentioned,) are firmly bound down to the transparent cornea, and consequently are not provided with the same resources against pressure from a column of blood; hence, when this fluid is suddenly and with impetuosity propelled into the eye, the vessels of the albuginea become turgid with blood, whilst whilst their continuations which pass over the cornea, not admitting of a sudden dilitation, sustain a very considerable degree of pressure and are violently stretched, not in their diameter but lengthways; if decided relief be not now given, the cornea very soon loses its vitality, it bursts, the humours of the eye are discharged, and eventually the loss of substance in the tunic, (which, being proportionable to the previous pressure, is extensive in this disease,) is renewed with opake lymph that never becomes vascular, and thus the eye, as the organ of sight, is irrecoverably lost.

Another less hopeless effect of the Egyptian ophthalmia, is a permanent state of chemosis, with vascular cornea, with or without opacity from extravasated lymph; this sort of case admits of considerable relief, as every person who has attended any of the eye institutions of the metropolis must have witnessed; here the violent excitement of the disease has produced an atony in the vessels of the eye, and particularly those of the conjunctiva, and the rational mode of cure, founded on the true principles of surgery, is first to destroy the vascularity of the cornea, by cutting through the diseased conjunctiva around the albuginea; and, secondly, with the combined view of contracting the vessels, of rousing the action of the absorbents, and of stimulating the entire eye, to apply to the organ powerful stimulants.

I trust I shall not be accused of judging rashly if I conclude that this is the sort of case, in the cure of which Mr. Adams's skill has been so remarkably conspicuous; nor will I insult the understanding of those gentlemen who wrote the paragraph in question, by supposing that they would designate such a disease the Egyptian ophthalmia; or that they are prepared to assert, that a violent inflammation of the eye, or of any organ of the body, could, for the space of three years, preserve its original character; as well might they affirm that a case of hydrothorax, supervening on acute inflammation of the chest, is still a case of pleuritis: but what is the inference which the public may have drawn from it? They have heard of and dreaded the Egyptian ophthalmia; they have been told that it has destroyed the eyes of hundreds of our brave soldiers, and, what more nearly concerns them, that the troops on their return to this country, brought hither the disease, where the contagion has ever since been kept up. All this will naturally crowd upon their recollection, when, on reading those official papers, they find that Sir William Adams has discovered a mode of curing that dreadful disease; and no doubt they will cordially agree with the medical officers of Greenwich Hospital, that the discovery is a great national desideratum.

The

The statement of the cases simply as it stands is conclusive on the merits of Sir William as an operator, and his success; but he claims for himself the merit of a new invention, and the operation of extraction is performed by him by a process totally different from the usual mode.

It must be evident to every one who has read his book, that he has designedly veiled it over with an air of pompous obscurity, whilst he excites the curiosity of the faculty even to wonder, by what rout he contrives to extract the diseased lens.

It should be recollected, that those operations on the larger members of the body, which modern surgeons perform so differently from their predecessors, are altered principally with the intention of simplifying the after-treatment, and counteracting the effects of the constitutional disease, which is always excited by the healing of large wounds. Amputation has undergone very considerable improvement, but has this rendered the operation more simple? Is it a method of taking away the useless limb with greater facility than before? or does it lessen the agony of the patient? All these interrogations must be answered in the negative, because the very reverse is the fact; for, if they were the desiderata of the operation, the old method of cutting directly down upon the bone, by a few sweeps of the knife, could not be improved upon. In this respect the operations on the eye, when the object is the extraction of a cataract, must always very materially differ; here the desideratum, to which all our improvements must principally be directed, is the extraction of the diseased lens, that is, the extirpation of the existing evil, with the least possible injury to the eye; and not with the view of counteracting the effects of the operation, or lessening the extent of the artificial wound; these considerations, joined with the well-known fact, that the diseased lens can only be brought away with safety by one route, will readily account for the generally-received opinion, that extraction was not susceptible of any farther improvement, independent of the superiority which considerable manual dexterity gives to its possessor. In short, the operation may be begun under the most favourable circumstances, the necessary flap in the cornea may be dexterously made, the crystalline capsule may be torn and the lens dislocated, and still, just at the moment as the operation is about to terminate, the iris, as well as the entire eye, may become irritable, and so contract upon the dislocated lens as to render its escape, without the interference of art, impracticable. Under these circumstances the operator commonly makes pressure on the eye-ball, which,

 if if not done with the greatest circumspection, breaks down the cells of the vitreous humour, and thus forces out a portion of their contents, whilst the cataract sinks from his view, and either the eye is lost, or the operation fails for the present.

These being my sentiments on this operation, I felt much interest to know by what new process a cataract could be extracted, so as to differ from the usual mode of operating; nor could I doubt that whilst the knight claimed for himself a decided superiority to other oculists, he would have shewn that he possessed the candour for which medical men are so deservedly eminent, by laying open to the profession this new process.

The honour of introducing into general practice the operation by solution, is justly due to Mr. Saunders; and I will defy any man to bring about this curative process, and abandon that gentleman's mode of operating. Can it be admitted, because Sir William cuts up the diseased lens more than Mr. Saunders did, and thus, by being enabled to push considerable fragments into the anterior chamber, he exposes them more immediately to the solvent power of the aqueous humour? Nay, is it not still the operation of Mr. Saunders which he performs? I will boldly answer in the affirmative; both have precisely the same object in view, to destroy the vitality of the diseased lens, and to place it in such a situation, as to give to the aqueous humour an opportunity of acting upon it as a solvent. The only difference, which I can discover, between these gentlemen's modes of operation, amounts to this: Sir William does more at one time than Mr. Saunders thought it necessary to do.

Before I conclude this paper, (although it has already far exceeded the limits which I first intended,) I will venture to say a few words on what this new mode of extraction may probably be founded. Sir William, in one of the paragraphs of his letter, says, " I have ascertained a fact of great practical importance, which will in a great degree explain the general bad success of extraction as it is usually performed, namely, that the vitreous humour was in a state of dissolution nearly in one-half of the eyes on which I operated: this is a diseased change which can rarely be perceived before the performance of the operation, and which authors agree must occasion a total destruction of the eye whenever the cataract is extracted in the usual manner. In these cases I performed an operation different from any of the former."

It would be preposterous to place a literal construction on this quotation; the knight, it would then appear, performs an operation different to any he has mentioned, (which are by by extraction and solution,) because a diseased change has taken place in the eye, *but which change, singular to say, eludes discovery until the operation is performed.*

C. W. SMERDON,
*Member of the College of Surgeons.*

*Clifton, Bristol; Oct.* 29, 1814.

